# A physician-led medical emergency team increases the rate of medical interventions: A multicenter study in Korea

**DOI:** 10.1371/journal.pone.0258221

**Published:** 2021-10-07

**Authors:** Su Yeon Lee, Jee Hwan Ahn, Byung Ju Kang, Kyeongman Jeon, Sang-Min Lee, Dong Hyun Lee, Yeon Joo Lee, Jung Soo Kim, Jisoo Park, Jae Young Moon, Sang-Bum Hong

**Affiliations:** 1 Department of Pulmonary and Critical Care Medicine, Asan Medical Center, University of Ulsan College of Medicine, Seoul, South Korea; 2 Department of Internal Medicine, Ulsan University Hospital, University of Ulsan College of Medicine, Ulsan, Korea; 3 Division of Pulmonary and Critical Care Medicine, Department of Medicine, Samsung Medical Center, Sungkyunkwan University School of Medicine, Seoul, Korea; 4 Division of Pulmonary and Critical Care Medicine, Department of Internal Medicine, Seoul National University Hospital, Seoul National University College of Medicine, Seoul, Korea; 5 Division of Pulmonary and Critical Care Medicine, Department of Internal Medicine, Dong-A University College of Medicine, Busan, Korea; 6 Division of Pulmonary and Critical Care Medicine, Department of Internal Medicine, Seoul National University Bundang Hospital, Seongnam, Korea; 7 Division of Pulmonary and Critical Care Medicine, Department of Internal Medicine, Inha University Hospital, Inha University School of Medicine, Incheon, Korea; 8 Division of Pulmonology, Allergy and Critical Care Medicine, Department of Internal Medicine, CHA University, CHA Bundang Medical Center, Seongnam, Korea; 9 Division of Pulmonary and Critical Care Medicine, Department of Internal Medicine, Chungnam National University Hospital, Chungnam National University College of Medicine, Daejeon, Korea; Beaumont Hospitals: Beaumont Health, UNITED STATES

## Abstract

**Background:**

According to the rapid response system’s team composition, responding teams were named as rapid response team (RRT), medical emergency team (MET), and critical care outreach. A RRT is often a nurse-led team, whereas a MET is a physician-led team that mainly plays the role of an efferent limb. As few multicenter studies have focused on physician-led METs, we comprehensively analyzed cases for which physician-led METs were activated.

**Methods:**

We retrospectively analyzed cases for which METs were activated. The study population consisted of subjects over 18 years of age who were admitted in the general ward from January 2016 to December 2017 in 9 tertiary teaching hospitals in Korea. The data on subjects’ characteristics, activation causes, activation methods, performed interventions, in-hospital mortality, and intensive care unit (ICU) transfer after MET activation were collected and analyzed.

**Results:**

In this study, 12,767 cases were analyzed, excluding those without in-hospital mortality data. The subjects’ median age was 67 years, and 70.4% of them were admitted to the medical department. The most common cause of MET activation was respiratory distress (35.1%), followed by shock (11.8%), and the most common underlying disease was solid cancer (39%). In 7,561 subjects (59.2%), the MET was activated using the screening system. The commonly performed procedures were arterial line insertion (17.9%), intubation (13.3%), and portable ultrasonography (13.0%). Subsequently, 29.4% of the subjects were transferred to the ICU, and 27.2% died during hospitalization.

**Conclusions:**

This physician-led MET cohort showed relatively high rates of intervention, including arterial line insertion and portable ultrasonography, and low ICU transfer rates. We presume that MET detects deteriorating patients earlier using a screening system and begins ICU-level management at the patient’s bedside without delay, eventually preventing the patient’s condition from worsening and transfer to the ICU.

## Introduction

The rapid response system (RRS) was first introduced in Australia and the United States in the mid-1990s [1]. Since the Institute for Healthcare Improvement’s “5 Million Lives” campaign recommended the implementation of the RRS, the RRS has been introduced and applied worldwide [2]. Currently, several hospitals in Korea are operating the RRS, and the quality and specialty of the RRS has improved [3,4].

The RRS aims to prevent unexpected patient death and cardiac arrest resulting from unpredictable deterioration of the patient’s condition in the general ward and emergency room. The RRS is composed of two limbs—an afferent limb that can quickly recognize a patient’s sudden deterioration and risk situation and an efferent limb that triages patients, performs resuscitation, and stabilizes the patients at the bedside [5]. Mainly, the efferent limb of the RRS includes the following interventions for resuscitation: therapy prescription, advanced airway management, central vascular line establishment, and intensive care unit (ICU)-level care initiation at the bedside [6]. Each country has different RRS types. According to team composition, responding teams were named as the rapid response team (RRT), medical emergency team (MET), and critical care outreach (CCO) [3]. RRTs and CCO are often nurse-led, whereas the MET is a physician-led team that mainly plays the role of an efferent limb of the RRS [3,5]. According to the MERIT study published in 2005, the introduction of the MET did not reduce the incidence of cardiac arrests, unplanned ICU admissions, or unexpected deaths [7]. In several subsequent studies, conflicting findings regarding the effectiveness of the RRS were reported [3,8–10].

Currently, more than 10 years later, various types of the RRS are operating worldwide, and the RRS is well established in many hospitals. As few recent studies have focused on METs, and most RRSs were physician-led METs in Korea, in this study, we retrospectively analyzed cases for which physician-led METs were activated. Data on subjects’ characteristics, activation causes, activation methods, outcomes, and interventions by the MET through a cohort from nine tertiary teaching hospitals were collected and reviewed.

## Materials and methods

### Study subjects

This multicenter retrospective cohort study included subjects over 18 years of age who were admitted to general wards in 9 tertiary hospitals in Korea from January 2016 to December 2017. Only the first MET activation during each hospitalization was included. If a MET directly reviewed the patient’s medical record, made a clinical decision, or treated the patient, it was judged as MET activation. Subjects without information regarding in-hospital mortality were excluded from the analysis.

### Medical emergency team

Each hospital’s MET operated differently from each other. Among the 9 hospitals, 3 operated their METs 24 h a day, 7 days a week, whereas 6 operated their METs part-time—2 hospitals operated their METs during regular working hours on weekdays, another 2 hospitals operated their METs extending to night hours on weekdays, and the remaining 2 hospitals operated their METs extending to the weekend. The MET was activated using a screening or calling system. The screening criteria of the nine hospitals were slightly different. Six hospitals used a single-parameter trigger system, two hospitals used the Modified Early Warning Score or National Early Warning Score (NEWS), and one hospital used a single-parameter trigger system along with NEWS. A single parameter, including abnormal blood pressure, high or low respiratory rate, tachycardia or bradycardia, fever or hypothermia, oxygen desaturation on pulse oximetry, neurological deterioration, threatened airway, lactic acidosis, hypoxemia, hypercapnia, and metabolic acidosis on arterial blood gas analysis, can also trigger the MET. The parameters were collected from the electronic medical charts of all subjects. Furthermore, the triggering threshold was slightly different among the hospitals (see S1 File). A call from a nurse or doctor who perceived that a patient’s condition is deteriorating at the bedside could also activate the MET. In seven hospitals, the MET could be activated by cardiopulmonary cerebral resuscitation (CPCR) situations. In eight hospitals, the MET included one or more doctors and nurses, and in one hospital, two doctors were included in the MET.

### Data collection

After the MET activation, a doctor or nurse in the activated team completed a standardized sheet related to the activation event. The following data were included: age, sex, date of admission, department of admission, diagnosis at admission, comorbidities, date and time of MET activation, interval between admission and MET activation, clinical department at the time of activation, method of activation, cause of activation, do-not-resuscitate (DNR) order before and after MET activation, MET interventions, and results of MET activation. The types of activation methods were screening, doctor call, nurse call, CPCR, and others (paramedics call), and the causes of activation were sepsis, shock, respiratory distress, cardiopulmonary arrest, arrhythmia, altered mental status, metabolic acidosis, and others. In addition, the ICU discharge date, hospital discharge date, and death date in subjects with MET activation were collected. The data extraction time (time zero) was based on the MET activation time in the medical record, and if there was no recorded value at the time of MET activation, the MET on-site arrival time was used. In the absence of both data, the recorded value of the closest point among the recorded values between 24 h before and after MET activation was collected. Interventions directly performed by the MET were collected.

### Statistical analysis

Variables were presented as either mean with standard deviation or median with an interquartile range (IQR), as appropriate. We compared the subjects who died with those who survived after MET activation; Student’s t-test was used to compare continuous variables, and the chi-squared test or Fisher’s test was used to compare categorical variables. All p values were two-tailed, and p values of less than 0.05 were considered statistically significant. Statistical Package for the Social Sciences (version 25.0; IBM Corp., Armonk, NY, USA) was used for all statistical analyses.

### Ethical statement

The study protocol was reviewed and approved by the Institutional Review Board of the National Cancer Center (IRB #2018–0181), which waived the requirement for informed consent because of the retrospective nature of this study.

## Results

Among the 12,803 cases of MET activation, 12,767 cases were analyzed in this study, excluding 36 cases without information regarding in-hospital mortality. Among the 12,767 cases, 9,299 survived and 3,468 died. The median age of the subjects was 67 years, and 58.7% were males (Table 1). Subsequently, 70.4% of the subjects were admitted to the medical department, and 29.6% were admitted to the surgical department. The most common underlying diseases were solid cancer (39%), diabetes mellitus (27.1%), and cardiovascular disease (23.4%). The median interval from admission to MET activation was 5 days. The characteristics of the subjects who died during hospitalization and those who survived after MET activation were compared. The subjects who died were more likely to be male (62.6% vs. 57.2%), were frequently admitted to the medical department (87.3% vs. 64%), and had more comorbidities, including solid cancer (48.2% vs. 35.6%), hematological malignancy (15.5% vs. 7.3%), chronic lung disease (15.7% vs. 13.8%), and hepatobiliary pancreatic disease (12.8% vs. 10.6%). The median interval from admission to MET activation was longer (8 days vs. 4 days) in subjects who died. The NEWS at the time of MET activation was higher in patients who died (8.9 ± 3.2 vs. 7.0 ± 3.3, p < 0.001). Other physiological parameters at the time of MET activation are presented in S1 Table.

**Table 1:** Subject characteristics.

Variable	Total	Survived	Died	p value
**Subjects, n**	12767	9299	3468	
**Age, year, median (IQR)**	67 (57–76)	67 (56–76)	67 (57–76)	.14
**Male sex, n (%)**	7489 (58.7)	5319 (57.2)	2170 (62.6)	< .001
**Department, n (%)**				
** Medical**	8984 (70.4)	5955 (64.0)	3029 (87.3)	< .001
** Surgical**	3783 (29.6)	3344 (36.0)	439 (12.7)	< .001
**BMI, mean ± SD**	22.4 ± 4.3	22.4 ± 4.3	22.3 ± 4.2	.33
**Comorbidities, n (%)**				
** Solid cancer**	4981 (39.0)	3308 (35.6)	1673 (48.2)	< .001
** Hematological malignancy**	1215 (9.5)	679 (7.3)	536 (15.5)	< .001
** Chronic lung disease**	1825 (14.3)	1279 (13.8)	546 (15.7)	.004
** Cardiovascular disease**	2992 (23.4)	2162 (23.2)	830 (23.9)	.42
** Hepatobiliary pancreatic disease**	1435 (11.2)	990 (10.6)	445 (12.8)	.001
** Gastrointestinal disease**	487 (3.8)	364 (3.9)	123 (3.5)	.34
** Cerebrovascular disease**	1508 (11.8)	1199 (12.9)	309 (8.9)	< .001
** Chronic kidney disease**	1371 (10.7)	1019 (11.0)	352 (10.1)	.19
** Thyroid disease**	407 (3.2)	317 (3.4)	90 (2.6)	.02
** Diabetes mellitus**	3458 (27.1)	2553 (27.5)	905 (26.1)	.12
** History of transplantation**	458 (3.6)	326 (3.5)	132 (3.8)	.42
**Days since admission, median (IQR)**	5 (2–13)	4 (1–11)	8 (2–19)	< .001

Abbreviations: BMI, body mass index; IQR, interquartile range; SD, standard deviation.

Concerning the activation method, 59.2% of the subjects were activated by a screening system, 25.2% by a doctor’s call, 11.7% by a nurse’s call, and 3.7% by CPCR announcement (Table 2). In the group of subjects who died after MET activation, the MET was less likely to be activated by a screening system (54.5% vs. 61.0%) and more likely to be activated by CPCR announcement (8.4% vs. 1.9%). In all subjects, common causes of MET activation were respiratory distress (35.1%), followed by shock (11.8%), and arrhythmia (6.1%), and in subjects who died, MET activation was more likely to be due to respiratory distress (45% vs. 31.4%), cardiopulmonary arrest (8.0% vs. 1.2%), and metabolic acidosis (5.9% vs. 3.4%).

**Table 2:** Medical emergency team activation methods and causes.

	Total (n = 12767)	Survived (n = 9299)	Died (n = 3468)	p value
**Activation methods, n (%)**				< .001
** Screening**	7561 (59.2)	5671 (61.0)	1890 (54.5)	
** Doctor’s call**	3214 (25.2)	2346 (25.2)	868 (25.0)	
** Nurse’s call**	1488 (11.7)	1088 (11.7)	400 (11.5)	
** CPCR announcement**	471 (3.7)	181 (1.9)	290 (8.4)	
** Others**	33 (0.3)	13 (0.1)	20 (0.6)	
**Causes, n (%)**				< .001
** Sepsis**	417 (3.3)	338 (3.7)	79 (2.3)	
** Shock**	1506 (11.8)	1088 (11.7)	418 (12.1)	
** Respiratory distress**	4485 (35.1)	2925 (31.4)	1560 (45.0)	
** Cardiopulmonary arrest**	385 (3.0)	108 (1.2)	277 (8.0)	
** Arrhythmia**	777 (6.1)	646 (6.9)	131 (3.8)	
** Altered mental status**	554 (4.3)	413 (4.4)	141 (4.1)	
** Metabolic acidosis**	518 (4.1)	315 (3.4)	203 (5.9)	
** Others**	4125 (32.3)	3467 (37.3)	658 (19.0)	

Abbreviations: CPCR, cardiopulmonary cerebral resuscitation.

The most common interventions were arterial line insertion (17.9%), intubation (13.3%), and portable ultrasonography (13.0%), and interventions were performed more in subjects who later died (Table 3). The MET performed interventions in 6,047 (47.4%) of the 12,767 subjects. The number of subjects who received more than 1 intervention was 297 subjects (71.2%) for sepsis, 1,115 subjects for shock (74%), 2,554 subjects (56.9%) for respiratory distress, 370 subjects (96.2%) for cardiopulmonary arrest, 248 subjects (31.9%) for arrhythmia, 344 subjects (62.1%) for altered mental status, and 246 subjects (47.5%) for metabolic acidosis. The most common causes of interventions were cardiopulmonary arrest, shock, sepsis, and altered mental status, in this particular order. Among the 6,047 subjects who received one intervention, the proportions of the interventions stratified according to activation causes are presented in Table 4. Arterial line insertion, intubation, portable ultrasonography, and intravenous vasopressor infusion were frequently performed, in this order, and particularly, arterial line insertion and portable ultrasonography were performed more regardless of the cause.

**Table 3:** Medical emergency team interventions.

Action	Total (n = 12767)	Survived (n = 9299)	Died (n = 3468)	p value
**ACLS**	515 (4.0)	143 (1.5)	372 (10.7)	< .001
**ECMO**	55 (0.4)	22 (0.2)	33 (1.0)	< .001
**Renal replacement therapy**	554 (4.3)	297 (3.2)	257 (7.4)	< .001
**Intubation**	1699 (13.3)	890 (9.6)	809 (23.3)	< .001
**BiPAP**	187 (1.5)	146 (1.6)	41 (1.2)	.11
**High-flow nasal cannula**	966 (7.6)	568 (6.1)	398 (11.5)	< .001
**Bronchoscopy**	163 (1.3)	118 (1.3)	45 (1.3)	.90
**Laryngoscopy**	41 (0.3)	22 (0.2)	19 (0.5)	.006
**Arterial line**	2281 (17.9)	1458 (15.7)	823 (23.7)	< .001
**Central line**	917 (7.2)	518 (5.6)	399 (11.5)	< .001
**Portable ultrasonography**	1661 (13.0)	1122 (12.1)	539 (15.5)	< .001
**CT scan**	1599 (12.5)	1141 (12.3)	458 (13.2)	.16
**IV antibiotics**	843 (6.6)	611 (6.6)	232 (6.7)	.81
**IV vasopressor**	1641 (12.9)	949 (10.2)	692 (20.0)	< .001
**Transfusion**	741 (5.8)	448 (4.8)	293 (8.4)	< .001
**DNR discussion**	1359 (10.6)	411 (4.4)	948 (27.3)	< .001

Abbreviations: ACLS, advanced cardiovascular life support; ECMO, extracorporeal membrane oxygenation; BiPAP, bilevel positive airway pressure; CT, computed tomography; IV, intravenous; DNR, do not resuscitate.

**Table 4:** Medical emergency team intervention rates according to the activation causes.

N (%)	Total (n = 6047)	Cardiopulmonary arrest (n = 370)	Shock (n = 1115)	Sepsis (n = 297)	Respiratory distress (n = 2554)	Altered mental status (n = 344)	Metabolic acidosis (n = 246)	Arrhythmia (n = 248)	Others (n = 873)	p value
**Arterial line**	2281 (37.7)	145 (39.2)	565 (50.7)	91 (30.6)	954 (37.4)	152 (44.2)	89 (36.2)	102 (41.1)	183 (21.0)	< .001
**Intubation**	1699 (28.1)	218 (58.9)	231 (20.7)	36 (12.1)	926 (36.3)	123 (35.8)	45 (18.3)	22 (8.9)	98 (11.2)	< .001
**Portable ultrasonography**	1661 (27.5)	108 (29.2)	385 (34.5)	149 (50.2)	587 (23.0)	96 (27.9)	74 (30.1)	73 (29.4)	189 (21.6)	< .001
**IV vasopressor**	1641 (27.1)	221 (6.1)	612 (54.9)	85 (28.6)	470 (18.4)	286 (57)	195 (4.1)	39 (15.7)	177 (20.3)	< .001
**CT scan**	1599 (26.5)	46 (12.5)	300 (26.9)	126 (42.6)	609 (23.8)	194 (56.4)	85 (34.6)	44 (17.7)	195 (22.4)	< .001
**High-flow nasal cannula**	966 (16.0)	1 (0.3)	61 (5.5)	10 (3.4)	741 (29.0)	7 (2.0)	16 (6.5)	29 (11.7)	101 (11.6)	< .001
**Central line**	917 (15.2)	80 (21.6)	298 (26.7)	49 (16.5)	280 (11.0)	46 (13.4)	68 (27.6)	19 (7.7)	77 (8.8)	< .001
**IV antibiotics**	843 (13.9)	10 (2.7)	195 (17.5)	79 (26.6)	348 (13.6)	38 (11.0)	21 (8.5)	59 (23.8)	93 (10.7)	< .001
**Transfusion**	741 (12.3)	55 (14.9)	299 (26.8)	32 (10.8)	142 (5.6)	42 (12.2)	30 (12.2)	26 (10.5)	115 (13.2)	< .001
**Renal replacement therapy**	554 (9.2)	31 (8.4)	110 (9.9)	23 (7.8)	162 (6.3)	46 (13.4)	85 (34.6)	14 (5.6)	83 (9.5)	< .001
**ACLS**	515 (8.5)	356 (96.2)	48 (4.3)	0 (0.0)	74 (2.9)	9 (2.6)	6 (2.4)	5 (2.0)	17 (1.9)	< .001
**BiPAP**	187 (3.1)	0 (0.0)	6 (0.5)	0 (0.0)	126 (4.9)	10 (2.9)	0 (0.0)	2 (0.8)	43 (4.9)	< .001
**Bronchoscopy**	163 (2.7)	5 (1.4)	6 (0.5)	1 (0.3)	114 (4.5)	7 (2.0)	3 (1.2)	2 (0.8)	25 (2.9)	.002
**ECMO**	55 (0.9)	24 (6.5)	14 (1.3)	0 (0.0)	13 (0.5)	1 (0.3)	0 (0.0)	1 (0.4)	2 (0.2)	< .001
**Laryngoscopy**	41 (0.7)	1 (0.3)	8 (0.7)	0 (0.0)	30 (1.2)	0 (0.0)	2 (0.8)	0 (0.0)	0 (0.0)	< .001

Abbreviations: ACLS, advanced cardiovascular life support; ECMO, extracorporeal membrane oxygenation; BiPAP, bilevel positive airway pressure; DNR, do not resuscitate; IV, intravenous; CT, computed tomography.

Among 12,767 subjects, 29.4% were transferred to the ICU after MET activation and stayed in the ICU for a median of 5 days (Table 5). The mean length of hospital stay after MET activation was 12 days, and the maximum length of hospital stay was 21 days. The subjects who eventually died were more likely to be transferred to the ICU (38.3% vs. 26.0%). According to MET activation causes, the ICU transfer rate was higher among subjects with shock (50.9%), altered mental status (39.9%), and respiratory distress (35.8%) (Table 6), although the in-hospital mortality was higher in subjects with metabolic acidosis (39.2%), respiratory distress (34.8%), and shock (27.8%).

**Table 5:** Outcomes of the medical emergency team activation.

Outcomes	Total (n = 12767)	Survived (n = 9299)	Died (n = 3468)	p value
**ICU transfer, n (%)**	3748 (29.4)	2421 (26.0)	1327 (38.3)	< .001
**Length of ICU stay, days, median (IQR)**	5 (3–10)	4 (3–9)	5 (2–12)	.053
**Post activation length of hospital stay, days, median (IQR)**	12 (5–25)	14 (7–27)	6 (2–19)	.01
**Length of hospital stay, days, median (IQR)**	21 (11–40)	21 (12–40)	20 (10–41)	< .001

Abbreviations: ICU, intensive care unit; IQR, interquartile range.

**Table 6:** ICU transfer rate and in-hospital mortality of patients for whom MET was activated according to activation causes.

	ICU transfer rate (%)	In-hospital mortality (%)
**Sepsis**	26.6	18.9
**Shock**	50.9	27.8
**Respiratory distress**	35.8	34.8
**Cardiac arrest**	62.9	71.9
**Arrhythmia**	21.2	16.9
**Altered mental status**	39.9	25.5
**Metabolic acidosis**	31.3	39.2
**Others**	11.5	15.9
**p value**	< .001	< .001

Abbreviations: ICU, intensive care unit.

## Discussion

Since MET has been introduced more than 10 years ago, we expect that the proficiency of METs has increased and its role in the hospital has been solidified. In addition, the performance of METs may have been improved along with the developments in medical technology and equipment. Furthermore, we thought that the characteristics of physician-led METs are different from other RRS types; thus, we compared the cohort in this study with those of other studies. In this cohort, most subjects were admitted to the medical wards and the most common cause of MET activation was respiratory distress, followed by shock. METs were activated by an electronic medical records-based screening system more than half of the time, and the commonly performed procedures were arterial line insertion, intubation, and portable ultrasonography at the bedside. Moreover, 29.4% of the subjects were transferred to the ICU, and 27.2% died while hospitalized.

The results of this study show higher intervention rates than those reported in other studies on the RRS. Above all, the interventions examined in this study excluded basic interventions, including fluid resuscitation, chest X-ray, arterial blood gas analysis, and medications other than intravenous antibiotics and vasopressors; therefore, the percentage of subjects who had received interventions more than once may be underestimated. However, the cohort in this study shows higher intervention rates when comparing by item. In the large-scale study by Lyons et al. that included 151,400 cases of RRT activation in major hospitals in the United States, the rate for intubation was 4.6% (vs. 13.3% in the cohort of this study), bedside ultrasonography was 0.3% (vs. 13% in the cohort of this study), and newly started antibiotics was 1.5% (vs. 6.6% in the cohort of this study), which were lower than those in this study [11]. In the study by Chan et al. published in 2008, the rate of intubation was 7.4% (vs. 13.3% in the cohort of this study), arterial line insertion was 0.8% (vs. 17.9% in the cohort of this study), and intravenous vasopressor administration was 1.3% (vs. 12.9% in the cohort of this study), which showed lower intervention rates than those reported in this study [12]. In addition, this study showed a higher intervention rate than other studies that mentioned the intervention rates of the RRS [13,14]. In the aforementioned studies, nurse-led RRTs were mainly focused on or it was unclear whether physicians were included in the responding team. However, in this study, all METs included physicians, and in many cases, the physician was an intensive care or pulmonology specialist skilled in resuscitations and interventions. Therefore, the physician had directly made a decision and performed diagnostic and therapeutic interventions, and for this reason, the necessary interventions had been performed in time without delay. In addition, technological equipment developments, such as video laryngoscopy or portable ultrasonography, have enabled physicians to easily perform interventions. Especially in this study, the rate of performing portable ultrasonography at patient bedside was higher, regardless of the activation causes. Performing bedside ultrasonography is beneficial in severely ill patients, as physicians evaluate the heart function and volume status in variable shock patients. Furthermore, ultrasonography-guided central line insertion increases the procedure’s success rate and accuracy and prevents procedural delays and complications, including pneumothorax and vascular injury.

Among the subjects in this study, 70.4% had been hospitalized in the medical department, which was a result similar to that reported in previous studies [11,15], but the proportions of subjects with solid cancer and hematological malignancy were high, at 39% and 9.5%, respectively, accounting for approximately half of all subjects (47.9%). However, the proportion of subjects with DNR discussion was 10.6%, and the ICU transfer rate was 29.5%, which were not higher than those reported in other studies [16,17]. This study showed a relatively lower ICU transfer rate than studies that showed a lower intervention rate than this study—the reported ICU transfer rate was 30% in the study by Lyons et al., 68.81% in that by Al-Omari et al., and 41.2% in that by Chan et al [12–14]. A higher intervention rate but lower ICU transfer rate could mean that a MET reduces ICU admission by promptly beginning ICU-level management at the patient’s bedside. In this study, more than 50% of the MET activation cases were triggered by the screening system, and among these, the ICU transfer rate was 19%; among the cases of MET activation by calling, the ICU transfer rate was 42.6%. That is, the MET’s screening system could also reduce patients’ ICU transfer rate. Hence, further study on whether MET activation by screening reduces the mortality or the prevalence of cardiac arrest is warranted.

In our cohort, in-hospital mortality among the 12,767 patients was 27.2%, which is similar to the mean value of 26% (range 12%–60%) in a systematic review of 17 studies that reported the in-hospital mortality of RRT activation cases [16]. Among the total cases, 1,055 cases had sepsis or septic shock: 417 cases had sepsis and 638 cases had septic shock. In 19% of the cases, antibiotics were newly started or changed by the MET. According to the results of the systematic review and meta-analysis by Vincent et al. published in 2019, the in-hospital mortality in patients with septic shock was 39% and 28/30-day mortality was 36.7% [18], and according to the study by Bauer et al. published in 2020, the 30-day mortality in patients with septic shock was 34.7% [19]. In this study, the in-hospital mortality in patients with sepsis and septic shock was 28.4% and the 28-day mortality was 22.9%, and in patients with septic shock only, the in-hospital mortality was 34.6% and the 28-day mortality was 27.7%. This study showed lower hospital mortality and 28-day mortality than the two aforementioned systematic reviews. According to the study by Arabi et al., after the introduction of an electronic alert system and sepsis response team, the in-hospital mortality in patients with sepsis and septic shock, mechanical ventilation, the length of ICU stay, and the length of hospital stay reduced [20]. This is associated with earlier detection of sepsis and increased compliance of the sepsis resuscitation bundle. According to a study by Sebat et al. [21], the mortality rate in patients with shock decreased from 40% to 11.8% 5 years after the introduction of the RRS and the mortality rate in patients with septic shock decreased from 50% to 10%. As with the one-bundle plan of the Surviving Sepsis guidelines [22], especially in the case of septic shock, early management has a decisive effect on the prognosis. Therefore, in this study, it is expected that early resuscitation and early goal-directed therapy by a skilled physician in an MET decrease in-hospital mortality in patients with sepsis and septic shock.

This study has some limitations. First, we investigated the causes of MET activation at the time of MET acting, so we did not account for the subjects’ additional identified problems or diagnosis after being transferred to the ICU. We included subjects with septic shock based on sepsis II definitions. Second, this study was not a before-and-after study—we did not have any comparable data before MET introduction. As we did not know the in-hospital mortality or ICU transfer rate before MET introduction, we compared our results with those reported in other studies instead. Third, this study was conducted in nine tertiary teaching hospitals; therefore, the subjects had several comorbidities and more severe disease than those in nonteaching, nontertiary hospitals. Therefore, the results may not be representative of all hospitals. Nevertheless, this study is meaningful because it demonstrated the well-established physician-led MET in Korea and is a model for other hospitals that are attempting to introduce the RRS.

## Conclusions

The results of this study confirmed high intervention and low ICU transfer rates. We presume that our MET detects the deterioration of the condition of patients earlier by a screening system and begins ICU-level management at the patient’s bedside without delay, eventually preventing the patient’s condition from worsening and transfer to the ICU.

## Supporting information

S1 File Medical emergency team activation criteria of each center. (DOCX)

S1 Table Physiological parameters and severity at the time of MET activation. (DOCX)
