# Supplementary material for: A physician-led medical emergency team increases the rate of medical interventions: A multicenter study in Korea
Source: PLoS One. 2021 Oct 7;16(10):e0258221. doi: 10.1371/journal.pone.0258221 (PMC8496774; doi:10.1371/journal.pone.0258221)
Supplement: S1 File — (DOCX) [file pone.0258221.s001.docx]

**Medical emergency team activation criteria of each center**

1. Seoul National University Hospital

A. Calling criteria

- Respiratory: respiratory rate ≤8 or ≥28, SpO_2_ ≤90% for 5 minutes, dyspnea of sudden onset

- Cardiovascular: heart rate ≤40 or ≥130, systolic blood pressure ≤80 or ≥200, systolic blood pressure 80–90 with symptom, chest pain not responsive to sublingual nitroglycerin

- Neurologic: altered consciousness of sudden onset, sudden paralysis of face or extremities, new onset seizure, prolonged agitation (≥10 minutes) not fully explained by medical conditions

- Others: color change of peripheral extremities, subjective judgement of attending physician or nurse

B. Screening criteria

- Heart rate: <41, >129

- Respiratory rate: <9, >27

- Systolic blood pressure: <81, >199

- SpO_2_: <90%

2. Seoul National University Bundang Hospital

- Systolic blood pressure: <90

- Heart rate: <50, >140

- Respiratory rate: <10, >30

- Body temperature: <36°C, >39°C

- SpO_2_: <90%

- ABGA: pH <7.25, PaCO_2_ >50, PaO_2_ <55

- Lactate >4 mmol/L

- tCO_2_ <15 mmol/L

3. Asan Medical Center

A. Calling criteria

- Airway: threatened, stridor

- Breathing: respiratory rate <6 or >30, SpO_2_ <90% on Venturi 40% or O_2_ 6 L/min

- Circulation: heart rate <40 or >140, systolic blood pressure <90

- Neurology: sudden mental change, seizure

- Others: bedside nurse's concern about overall deterioration

B. Screening criteria

- Systolic blood pressure <86

- Sudden mental change or unexplained agitation

- Applying O_2_ >9 L/min, or FiO_2_ >35%

- Respiratory rate >27 or <8

- Unexplained heart rate >140 or <40

- Unexplained severe metabolic acidosis: pH <7.3, lactate >2 mmol/L, tCO_2_ <12 mmol/L

- PaCO_2_ >50 or PaO_2_ ≤55

- Bedside nurse concern about overall deterioration

4. Samsung Medical Center

A. Calling criteria

- Respiratory: respiratory rate ≥30, SpO_2_ <85% for 5 minutes, pH <7.3 & PaCO_2_ >50, stridor, use of accessary respiratory muscles

- Cardiovascular: systolic blood pressure <85, heart rate >130, acute chest pain, symptomatic arrhythmia

- Neurologic: altered mental status of sudden onset, unexplained agitation, seizure

- Others: bedside nurse concern about overall deterioration

B. Screening criteria

- MEWS (modified early warning score) ≥7

5. Chungnam National University Hospital

A. Calling criteria

- Airway: airway obstruction sign, stridor

- Breathing: respiratory rate <8 or >30, pH <7.3 & PaCO_2_ >60, SpO_2_ <90% on facial mask or high flow nasal cannula

- Circulation: heart rate <40 or >140, systolic blood pressure <90, lactate >2 mmol/L

- Neurology: sudden mental change or unexplained agitation, seizure

- Nurse’s concern about overall deterioration

B. Screening criteria

- based on NEWS (national early warning score)

6. Inha University Hospital

A. Calling criteria

- Breathing: respiratory rate ≤8 or ≥30, SpO_2_ <90% for 5 minutes on O_2_ >5 L/min, PaCO_2_ >50

- Circulation: systolic blood pressure <90, heart rate <40 or >130, acute chest pain

- Neurology: sudden mental change, seizure

- Others: bedside nurse’s concern about overall deterioration

B. Screening criteria

- Breathing: respiratory rate ≤8 or ≥25, SpO_2_ ≤90%, PaO_2_ ≤55, PaCO_2_ ≥50, pH ≤7.3, lactate ≥2 mmol/L, tCO_2_ ≤12 mmol/L

- Circulation: systolic blood pressure ≤90, heart rate ≤40 or ≥130, acute chest pain

- Neurology: sudden mental change, seizure

- 48 hours after discharge from ICU

- High risk surgical patients

7. Ulsan University Hospital

A. Calling criteria

- Airway: threatened airway, stridor, wheezing sound

- Breathing: respiratory rate ≤6 or ≥30, SpO_2_ <90% on O_2_ 6 L/min or venturi mask 40%

- Circulation: heart rate <40 or ≥140, systolic blood pressure <90

- Neurology: sudden mental change, seizure

- Others: bedside nurse’s concern about overall deterioration

B. Screening criteria

- Airway: threatened airway, stridor, wheezing sound

- Breathing: respiratory rate ≤6 or ≥30, SpO_2_ <90%, oxygen demand (over nasal prong 6 L/min or venturi mask 40%)

- Circulation: heart rate <40 or ≥140, systolic blood pressure ≤85

- Neurology: sudden mental change, seizure

- Others: pH ≤7.33, HCO_3_ ≤15 mmol/L, lactate ≥2 mmol/L, potassium ≥6 mmol/L, tCO_2_ ≤15 mmol/L, glucose ≤50 mg/dL, ICU discharge patients

8. Dong-A University Hospital

- respiratory rate >28 or <8

- heart rate >140 or <40

- systolic blood pressure <90

- ABGA: pH <7.3 or >7.6, HCO_3_ <14 mmol/L, PaCO_2_ >50, PaO_2_ <55

- Lactate >30 mg/dL, tCO_2_ <15 mmol/L, potassium >6 or <3 mmol/L

- SpO_2_ <90%, O_2_ >8 L/min

9. CHA Bundang Medical Center

- mean blood pressure <60 or systolic blood pressure <90

- applying O_2_ >9 L/min or venturi mask >35%

- respiratory rate >25 or <8

- heart rate >140 or <40

- glucose ≤50 mg/dL

- unexplained severe metabolic acidosis: pH<7.3 or lactate >2 mmol/L or tCO_2_ <16 mmol/L

- PaO_2_ ≤55 or PaCO_2_ >50

- sudden mental change or unexplained agitation, unexplained seizures

- chest pain, upper airway obstruction sign like stridor

- bedside nurse concern about overall deterioration
