# Supplementary material for: A physician-led medical emergency team increases the rate of medical interventions: A multicenter study in Korea
Source: PLoS One. 2021 Oct 7;16(10):e0258221. doi: 10.1371/journal.pone.0258221 (PMC8496774; doi:10.1371/journal.pone.0258221)
Supplement: S1 Table — (DOCX) [file pone.0258221.s002.docx]

**Table S1. Physiological parameters and severity at the time of MET activation.**

| Variables | Total (n = 12767) | Survivor (n = 9299) | Died (n = 3468) | p value |
| --- | --- | --- | --- | --- |
| Systolic blood pressure, mmHg, mean ± SD | 113.1 ± 33.6 | 114.1 ± 32.5 | 110.6 ± 36.2 | .006 |
| ≤ 90, n (%) | 3487 (27.4) | 2973 (27.7) | 914 (26.4) | <.001 |
| ≥ 200, n (%) | 101 (0.8) | 85 (0.9) | 16 (0.5) | .01 |
| Heart rate, beats/min, mean ± SD | 102.8 ± 29.9 | 101.4 ± 28.6 | 106.4 ± 32.7 | .002 |
| ≤ 40, n (%) | 273 (2.1) | 121 (1.3) | 152 (4.4) | <.001 |
| ≥ 130, n (%) | 2293 (18.0) | 1520 (16.4) | 773 (22.4) | <.001 |
| Respiratory rate, breaths/min, mean ± SD | 23.3 ± 7.3 | 22.9 ± 6.8 | 24.4 ± 8.5 | <.001 |
| ≤ 8, n (%) | 219 (1.7) | 79 (0.9) | 140 (4.1) | <.001 |
| ≥ 25, n (%) | 4152 (32.7) | 2664 (28.8) | 1488 (43.2) | <.001 |
| Body temperature, ℃, mean ± SD | 37.1 ± 0.9 | 37.2 ± 0.9 | 37.0 ± 0.8 | <.001 |
| ≤ 36.0, n (%) | 719 (5.7) | 492 (5.3) | 227 (6.7) | .006 |
| ≥ 38.0, n (%) | 2167 (17.2) | 1641 (17.8) | 526 (15.5) | .001 |
| Mental status, n (%) |  |  |  | <.001 |
| Alert | 8585 (69.6) | 6649 (73.8) | 1936 (58.3) |  |
| Confused | 609 (4.9) | 384 (4.3) | 225 (6.8) |  |
| Responsive to voice | 1510 (12.2) | 1014 (11.2) | 496 (14.9) |  |
| Responsive to pain | 730 (5.9) | 477 (5.3) | 253 (7.6) |  |
| Unresponsive | 899 (7.3) | 491 (5.4) | 408 (12.3) |  |
| SpO_2_, %, mean ± SD | 92.2 ± 10.2 | 92.8 ± 8.9 | 90.7 ± 12.7 | <.001 |
| ≤ 91, n (%) | 3651 (30.5) | 2429 (28.2) | 1222 (36.6) | <.001 |
| Oxygen supply, n (%) | 7594 (59.5) | 5077 (54.6) | 2517 (72.6) | <.001 |
| SpO_2_/FiO_2_ ratio, mean ± SD | 318.3 ± 122.4 | 334.0 ± 117.7 | 277.9 ± 124.9 | <.001 |
| SpO_2_/FiO_2_ ratio, n (%) |  |  |  | <.001 |
| ≥ 357 | 4798 (40.1) | 3871 (44.9) | 927 (27.8) |  |
| < 357 | 4324 (36.1) | 3134 (36.3) | 1190 (35.6) |  |
| < 214 | 2676 (22.4) | 1546 (17.9) | 1130 (33.8) |  |
| < 89 | 164 (1.4) | 72 (0.8) | 92 (2.8) |  |
| NEWS, mean ± SD | 7.5 ± 3.4 | 7.0 ± 3.3 | 8.9 ± 3.2 | .007 |

Abbreviations: MET, medical emergency team; SD, standard deviation; SpO_2_, percutaneous oxygen saturation; FiO_2_, fraction of inspired oxygen; NEWS, National Early Warning Score
